# Additivity, Not Synergy, Underlies the Efficacy of Current Combination Regimens in Urothelial Cancer

**DOI:** 10.1158/2767-9764.CRC-26-0157

**Published:** 2026-06-19

**Authors:** Noah M. Schlachter, Eric J. Miller, Jonathan F. Anker, Matthew D. Galsky, Adam C. Palmer

**Affiliations:** 1 Department of Pharmacology, Computational Medicine Program, UNC Lineberger Comprehensive Cancer Center, University of North Carolina at Chapel Hill, Chapel Hill, North Carolina.; 2 Department of Medicine, Tisch Cancer Institute, Icahn School of Medicine at Mount Sinai, New York, New York.

## Abstract

PD-1/PD-L1 immune checkpoint inhibitor (ICI)-based combination regimens have transformed metastatic urothelial cancer treatment. However, phase III trials of these regimens have shown varied results. We sought to understand these differences by assessing whether these combinations are synergistic, additive, or less-than-additive, which is crucial for translational research and clinical development. We systematically identified five randomized phase III trials of ICI-based combinations for metastatic urothelial carcinoma and tested whether progression-free survival (PFS) distributions of combinations were as predicted by a null hypothesis of additivity, defined as the sum of PFS durations of individual drugs. For KEYNOTE-361 and IMvigor130, which allowed cisplatin or carboplatin, we evaluated platinum-specific predictions. We found that PFS distributions in trials adding PD-1/PD-L1 ICI to enfortumab vedotin (EV-302), gemcitabine plus cisplatin (CheckMate 901), or CTLA-4 blockade (DANUBE) were statistically indistinguishable from additivity. In trials using choice of cisplatin or carboplatin (KEYNOTE-361, IMvigor130), ICI combinations with cisplatin–gemcitabine yielded additive PFS, whereas ICI combinations with carboplatin–gemcitabine yielded less-than-additive PFS, indicating that carboplatin blunts the potential benefits of PD-1/PD-L1 inhibitors. The predictable success of EV-302 and CheckMate 901 demonstrate that substantial improvements in clinical outcomes can be achieved by additive combinations of individually effective agents. Overall, modeling PFS from single-agent data could have anticipated most contemporary phase III trial outcomes, potentially informing rational trial design and prioritization.

**Significance:**

Practice-changing PD-1/PD-L1 immune checkpoint combinations in urothelial cancer show additive, not synergistic, efficacy in phase III trials. However, carboplatin was significantly less-than-additive with immunotherapy, helping explain negative results of KEYNOTE-361 and IMvigor130.

## Introduction

For decades, platinum-based combination chemotherapy remained the primary treatment for metastatic urothelial cancer, with little progress in developing new therapeutic options during this extended period (1–3). PD-1/PD-L1 immune checkpoint inhibitors (ICI) have recently transformed the treatment landscape of metastatic urothelial cancer, with durable responses achieved in 20% to 30% of patients (1, 4–6). These results prompted multiple phase III trials investigating whether combining PD-1/PD-L1 ICIs with other systemic therapies could improve first-line treatment of metastatic urothelial cancer. These trials have yielded mixed results, with some combinations failing to improve upon chemotherapy alone and others ushering in new treatment standards (Table 1). The reasons for these large differences in outcome have been unclear. With limited resources and an overwhelming number of potential combinations, there is a critical need to better understand *why* some combinations succeed in an effort to refine strategies that inform *how* to develop future combinations.

**Table 1.** Summary of phase III combination therapy trials in advanced urothelial carcinoma.

Trial	Patients enrolled (I/C)	Intervention 1	Intervention 2	Control	FDA approval
IMvigor130 Galsky and colleagues 2020	1,213 (451/362/400)	Atezolizumab plus gemcitabine and cisplatin or carboplatin, followed by atezolizumab maintenance	Atezolizumab alone	Gemcitabine plus cisplatin or carboplatin	N/A
DANUBE Powles and colleagues 2020	1,032 (346/342/344)	Durvalumab plus tremelimumab followed by durvalumab maintenance	Durvalumab alone	Gemcitabine plus cisplatin or carboplatin	N/A
KEYNOTE-361 Powles and colleagues 2021	1,010 (351/307/352)	Pembrolizumab plus gemcitabine and cisplatin or carboplatin, followed by pembrolizumab maintenance	Pembrolizumab alone	Gemcitabine plus cisplatin or carboplatin	N/A
Checkmate 901 Heijden and colleagues 2023	608 (304/304)	Nivolumab plus gemcitabine and cisplatin, followed by nivolumab maintenance	N/A	Gemcitabine plus cisplatin	2023
EV-302 Powles and colleagues 2024	886 (442/444)	Enfortumab vedotin plus pembrolizumab	N/A	Gemcitabine plus cisplatin or carboplatin	2024

Combination regimens in oncology are often motivated by the goal of synergy—where the efficacy of two drugs given together is greater than the sum of their individual effects. Clinical synergy is generally assumed to result from mechanisms of cellular, molecular, or immunologic interaction whereby the effects of one drug enhance the efficacy of another drug. Alternatively, combination regimens may succeed by having additive effects on time to disease progression. Synergy as exemplified by “1 plus 1 makes 3” does not guarantee greater efficacy than an additive combination achieving “10 plus 10 makes 20.” Indeed, additive efficacy is the anticipated consequence of overcoming intratumor and interpatient heterogeneity with combination therapy (7).

We recently developed a model to test whether the clinical efficacy of a drug combination is greater than, equal to, or less than what would be expected if the individual agents had numerically additive effects on time to disease progression. Rather than adding a summary statistic like median time, this model samples from the Kaplan–Meier distributions of each drug’s progression-free survival (PFS) times, simulating virtual patients whose PFS times for drug A and drug B are drawn with partial correlation. The model adds these times to progression—as in “5 months plus 5 months equals 10 months”—correcting for shared time to first scan to generate a PFS distribution expected from additive PFS times. Combination therapies have complex molecular effects, and this model tests whether such complexities result in longer PFS than expected from adding each drug’s individual effect on time to progression. We applied this model to analyze combination therapies that were FDA-approved for advanced cancer between 1995 and 2020, which spanned 14 cancer types and 49 anticancer agents, and found that 95% of approved regimens elicited additive or less-than-additive time to disease progression (8). Though some regimens were more or less effective than expected, every successful regimen was categorically expected to succeed based upon addition of single-agent activities. A sizable portion of combinations (27%) were significantly inferior to additivity, indicating that adverse interactions can meaningfully diminish clinical efficacy (8).

Randomized trials commonly quantify the relative effectiveness of treatments by a hazard ratio (HR), and one could consider modeling the effect of combination therapy “A + B” by multiplying the HRs of A and B relative to a control. However, HR is not an intrinsic measure of a drug’s efficacy but a comparison with some other control treatment (neither A nor B). Consequently, multiplying HRs yields different predictions for A + B depending on past control treatments, even for the same absolute effects of A and B (Supplementary Fig. S1). In contrast, summing PFS durations of A and B depends only on the effects of A and B. For this reason, adding absolute effects—times to progression—yields a specific, reproducible prediction, whereas multiplying relative benefits does not.

Five randomized phase III trials of ICIs in first-line advanced urothelial cancer have reported results in recent years. KEYNOTE-361 tested the PD-1 inhibitor pembrolizumab, and IMvigor130 the PD-L1 inhibitor atezolizumab, each combined with gemcitabine–platinum with investigator’s choice of cisplatin or carboplatin (9, 10). Neither trial met coprimary endpoints of PFS and overall survival (OS), but subset analyses in both trials suggested greater benefit of PD(L)-1 inhibition with cisplatin than with carboplatin (9, 10). Building upon that signal, CheckMate 901 evaluated the PD-1 inhibitor nivolumab with gemcitabine–cisplatin (not permitting carboplatin) and was the first chemo-ICI regimen to significantly improve both PFS and OS in this setting (11). In contrast, the DANUBE trial found that dual ICI therapy with the PD-L1 inhibitor durvalumab and the CTLA-4 inhibitor tremelimumab did not outperform chemotherapy (12). Finally, EV-302 tested pembrolizumab plus the antibody–drug conjugate enfortumab vedotin, which reduced the hazard for progression by 55% and the hazard for death by 53%—a remarkable advance in the treatment of advanced urothelial cancer (13).

With many completed, ongoing, and forthcoming trials of combination therapy in advanced urothelial cancer, determining which regimens owe their success—or failure—to synergistic, additive, or less-than-additive effects has potentially profound implications: (i) if additivity predominates, combining the most effective non–cross-resistant single agents may be the optimal strategy to enhance clinical outcomes; (ii) drug interactions may inform which cytotoxic agents combine most favorably with ICIs; and (iii) the success or failure of new combination therapies could be predicted before launching phase III trials by models based on individual drugs’ efficacies.

In this study, we analyze these five completed phase III combination therapy trials leveraging ICIs in the treatment of metastatic urothelial carcinoma and test whether their PFS results reject the null hypothesis of additive efficacy; that is, were their results predictable from the principle of drug additivity? Additionally, trials of ICIs with platinum-based chemotherapy have suggested that survival benefits may differ between cisplatin or carboplatin backbones. We therefore perform separate analyses for ICIs paired with cisplatin or with carboplatin to assess whether drug interactions differ between choice of platinum.

## Materials and Methods

### Data collection

**Figure 1.**
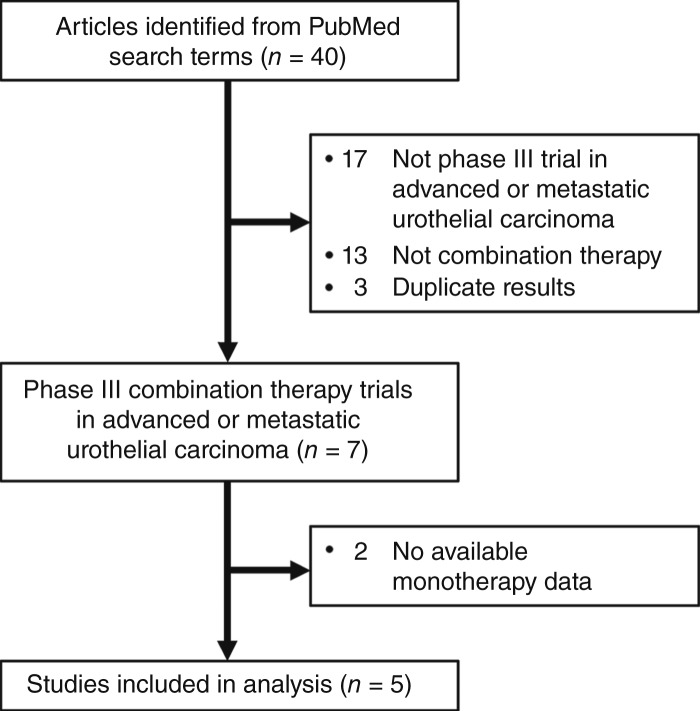
Flow diagram for selection of phase III trials of combination regimens in advanced urothelial carcinoma.

We conducted a structured literature search in PubMed to identify published phase III trials of combination therapy in advanced urothelial cancer since the first systemic immunotherapy approval for this disease in 2016 (Fig. 1). Search terms were (“Randomized Controlled Trial” [pt] and “phase 3” and (metastatic or advanced) and (“Urinary Bladder Neoplasms” [MAJR] or “Urologic Neoplasms” [MAJR]) and (bladder or urothelial)). Search terms revealed five randomized, phase III combination therapy regimens of ICIs in advanced or metastatic urothelial carcinoma, including IMvigor130, DANUBE, KEYNOTE-361, CheckMate 901, and EV-302 (Table 1). All trials were conducted on patients with previously untreated disease. We included all trials with published PFS distributions for combination therapy as well as for its constituent drugs (in which one “constituent” may be a combination such as gemcitabine plus platinum). For trials that tested treatments “A” versus “A + B” and did not study “B” only, we searched publications and conference abstracts reporting PFS data for treatment “B” in advanced urothelial cancer administered at the same or near-identical dose (Supplementary Fig. S2). Baseline characteristics (age, sex, race/ethnicity, performance status, nodal-only disease, and visceral metastases) were collected from each arm. Trials, treatments, and patient characteristics are in Supplementary Table S1 (6, 9–17). This search aimed to identify trials to be individually tested for non-additive PFS; because it is not a systematic review or meta-analysis that aggregates effects across trials, protocol registration was not applicable. Individual patient data (IPD) were imputed from digitized PFS curves and at-risk tables as previously described (18, 19). Kaplan–Meier PFS curves generated using imputed IPD are displayed alongside digitized PFS curves from trial publications in Supplementary Fig. S3A–S3T.

All data used in this study were derived from published phase II and phase III clinical trials (Supplementary Table S1). The original studies obtained written informed consent from all participants, were approved by the appropriate institutional review boards, and were conducted in accordance with recognized ethical guidelines, including the Declaration of Helsinki. No new human subjects research was performed for this analysis.

### Predicting PFS for combination regimens

The PFS distribution of a drug combination, under the null hypothesis of an additive effect, was calculated by adding PFS times of constituent drugs as previously described (8). Briefly, PFS distributions of single agents were represented as 10,000 discrete increments (each of 0.01%), and such distributions for drugs A and B were used to construct a partly correlated joint distribution. Points were sampled from the joint distribution, each containing two PFS times that represent a hypothetical patient’s response to the individual constituents of a drug combination. The two PFS times were added, minus the time of the first scheduled scan (approximately 2 months), because disease progression at or before the first scheduled scan is interpreted as “no benefit” rather than “2 months of benefit.” For each randomized trial, a HR expected from additivity was calculated by applying the Cox proportional hazards model to that trial’s actual control arm PFS and the expected combination therapy PFS. A graphical representation of this method is illustrated in Supplementary Fig. S4. Cox regression was used to determine whether there was a statistically significant difference between expected PFS under additivity and a trial’s observed combination arm PFS. Sources of PFS data for agents “A” and “B” are in Supplementary Table S1, with backbone “A” measured in previously untreated patients and added agent “B” measured in previously treated patients.

KEYNOTE-361 and IMvigor130 involved investigator’s choice of cisplatin- or carboplatin-based chemotherapy. To investigate whether drug interactions differ by choice of platinum agent, we tested for non-additivity, as above, using PFS data of gemcitabine and cisplatin to predict ICI plus gemcitabine and cisplatin and similarly to predict ICI plus gemcitabine and carboplatin.

## Results

We identified five phase III trials involving ICI-containing combination regimens in first-line treatment of metastatic urothelial cancer and for which PFS distributions were available for the combination therapy (“A plus B”) as well as the individual components (“A” and “B”, noting that the backbone “A” is sometimes itself a drug combination, e.g., gemcitabine plus cisplatin). Two trials could not be analyzed for lack of monotherapy data. Baseline patient characteristics were reasonably consistent across age, gender, ethnicity, lower-tract versus upper-tract disease, prevalence of lymph-node only disease, visceral metastases, and metastatic versus locally advanced disease where reported (Supplementary Table S1).

**Figure 2.**
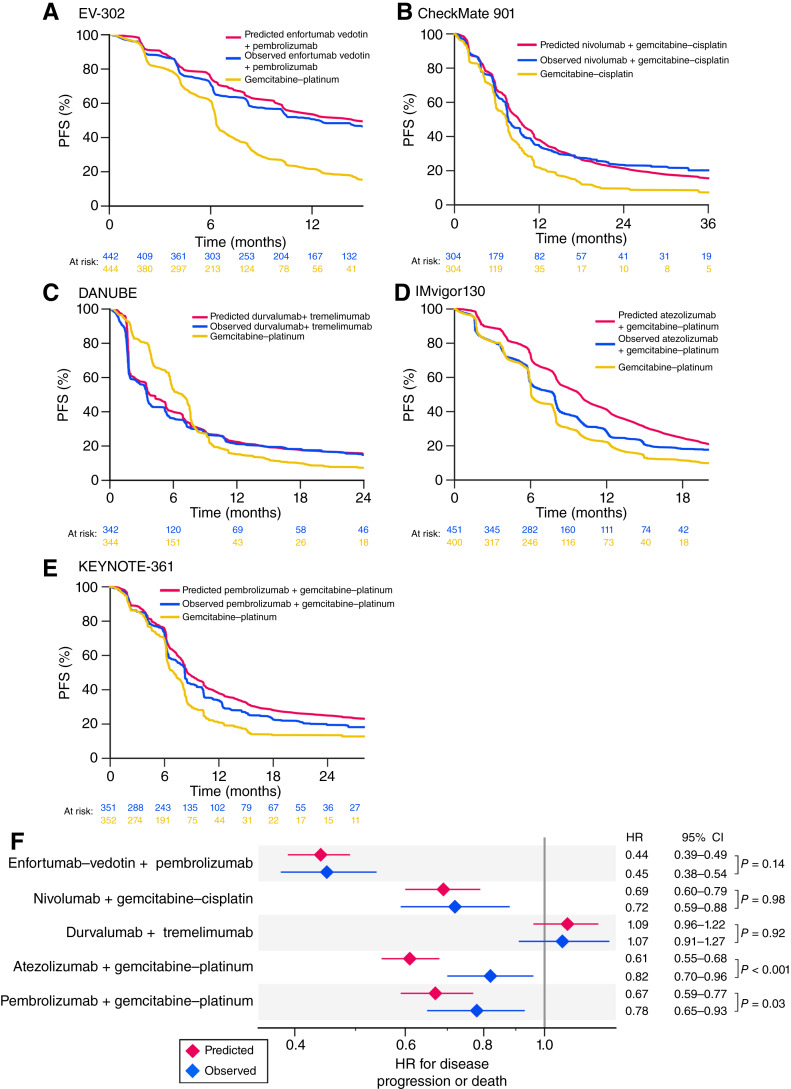
Observed and predicted PFS for phase III combination therapy trials in advanced urothelial carcinoma. PFS for combination therapies observed in clinical trials (blue) and as predicted by the additive effect of their constituents (red) for phase III trials in advanced urothelial carcinoma. The control arm of each trial is shown in yellow. Combination therapy data are from (**A**) EV-302, (**B)** CheckMate 901, (**C)** DANUBE, (**D)** IMvigor130, and (**E)** KEYNOTE-361; monotherapy data sources are in Supplementary Table S1. **F,** Forest plot of predicted (red) and observed (blue) hazard ratios for disease progression or death (Cox proportional hazards model), comparing each combination therapy with the control arm of its trial. *P* values obtained by comparing observed combination therapy PFS and additive predictions using the Cox proportional hazards model.

Observed and predicted PFS distributions are in Fig. 2A–E, and corresponding PFS HRs are in Fig. 2F. For three of five combination regimens, PFS distributions were statistically indistinguishable via Cox Regression from the sum of the PFS durations of their constituents, i.e., as predicted by additivity; these included approved regimens enfortumab vedotin plus pembrolizumab (*P* = 0.144) and nivolumab plus gemcitabine–cisplatin (*P* = 0.976), as well as durvalumab plus tremelimumab (*P* = 0.919) which was not superior to chemotherapy and so has not become a standard treatment in urothelial cancer. Phase II data on enfortumab vedotin plus pembrolizumab was also consistent with additivity (Supplementary Fig. S5). For two of five combination regimens, PFS distributions were significantly inferior to the sum of PFS durations of their constituents, i.e., less than additive; these regimens included platinum-based chemotherapy plus atezolizumab (*P* < 10^−6^) and platinum-based chemotherapy plus pembrolizumab (*P* = 0.025), neither of which significantly improved PFS. None of the combinations exhibited significantly more than additive PFS, i.e., synergistic.

**Figure 3.**
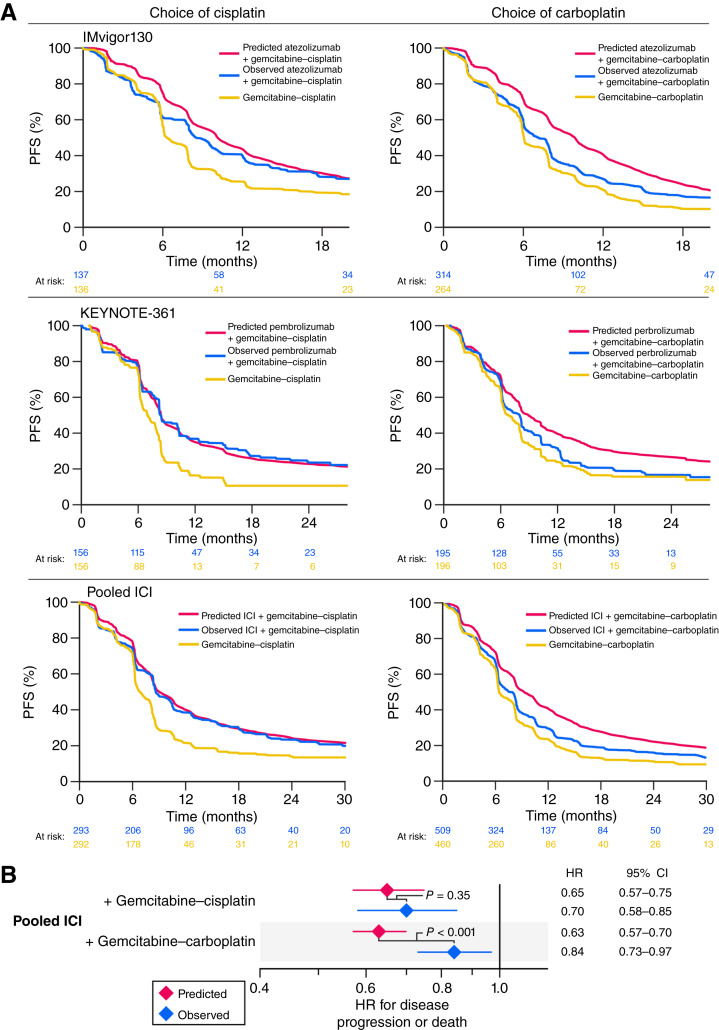
ICIs are additive with cisplatin but less-than-additive with carboplatin. **A,** PFS for combination therapies observed in clinical trials (blue) and as predicted by additivity (red), for choice of cisplatin and choice of carboplatin patients in IMvigor130 (top), KEYNOTE-361 (middle), and a pooled analysis of IMvigor130 and KEYNOTE-361 (bottom). For the pooled analysis, numbers at risk are imputed IPD. Predictions of combination therapy PFS for cisplatin-containing combinations were made using PFS for patients who received gemcitabine–cisplatin in IMvigor130 and KEYNOTE-361, and similarly for carboplatin. The control arm of each trial is shown in yellow. **B,** Forest plot of predicted (red) and observed (blue) HRs for disease progression or death (PFS; Cox proportional hazards) comparing ICI + chemotherapy vs. chemotherapy alone in a pooled analysis of choice of cisplatin (top) and choice of carboplatin (bottom) patients. Observed and predicted PFS did not significantly differ in choice of cisplatin patients (*P* = 0.35, Cox proportional hazards), but they differed significantly in choice of carboplatin patients (*P* < 0.001, Cox proportional hazards).

Adding PD-1/PD-L1 ICI to gemcitabine plus platinum chemotherapy improved survival in the CheckMate 901 study but not in the IMvigor130 and KEYNOTE-361 studies. A key difference between these studies was the choice of platinum: whereas IMvigor130 and KEYNOTE-361 allowed investigator’s choice of cisplatin or carboplatin, CheckMate 901 exclusively used cisplatin. To test whether choice of platinum alters the efficacy of ICI-containing regimens, we analyzed observed versus predicted PFS using data from IMvigor130 and KEYNOTE-361 stratified by choice of cisplatin or carboplatin. Whereas patients receiving combination therapy with cisplatin exhibited PFS distributions that were statistically indistinguishable from additive (consistent with CheckMate 901), patients receiving combination therapy with carboplatin exhibited PFS distributions significantly shorter than predicted (*P* < 0.001, Cox proportional hazards; Fig. 3A and B; Supplementary Fig. S6). In summary, we observe across multiple trials that cisplatin and carboplatin have categorically different interactions with ICIs in advanced urothelial carcinoma, with ICI plus cisplatin providing additive benefits to PFS durations and ICI plus carboplatin yielding significantly less-than-additive benefits to PFS durations.

While these combination therapy trials (“A + B”) occurred in the first-line setting, single-agent PFS data for the added drug (“B”) were often only available in the relapsed or refractory setting. It is unclear whether first-line data on the added drug are ideal or whether second-line data for the added drug (“B”) better reflects its capacity to improve efficacy by inhibiting tumor cells that resist the backbone of a combination (“A”). To determine whether use of first- or second-line ICI monotherapy data affects our results, we performed a sensitivity analysis on the KEYNOTE-361 trial, which is one of the few cases in which monotherapy PFS data for the added agent (pembrolizumab) is available in both settings, as KEYNOTE-361 included a pembrolizumab monotherapy arm (9, 20). Conclusions for the overall trial result as well as for cisplatin or carboplatin subsets were robust to the use of pembrolizumab monotherapy data from the first-line setting (KEYNOTE-361; Supplementary Fig. S7A and S7B) or second-line setting (KEYNOTE-045; Fig. 1E).

## Discussion

After decades of limited progress, ICI-based combination regimens have emerged as new first-line standards for metastatic urothelial cancer (11, 13). We sought to understand why these regimens improve outcomes to inform principles for the development of future therapies. We found that the magnitude of clinical benefit of approved ICI-based combination regimens, including enfortumab vedotin plus pembrolizumab, was quantitatively consistent with additivity. These results contradict the notion that synergy is needed to develop effective combination therapies, which has key implications for translational research and clinical drug development. Our findings underscore the critical need to develop single agents with novel mechanisms of action that have (i) single-agent activity, (ii) non-overlapping adverse event profiles, and (iii) are non–cross-resistant with current therapies.

“Rational” design of combination regimens typically seeks synergistic interaction, in which drugs enhance each other’s efficacy to produce more-than-additive effects. Indeed, the National Cancer Institute’s Clinical Trial Design Taskforce recommends that phase I trials of combination therapies have a *rationale for pharmacological or biological interaction* (21). This paradigm has been complicated by three observations. First, studies have demonstrated that preclinical synergy does not correlate with clinical success (22, 23). Second, effective combination therapies are often labeled synergistic without formal tests for more-than-additive effect. Consequently, belief in necessity of synergy has not been founded on evidence. Third, recent analysis of approved combination therapies across 25 years of FDA approvals found that a large majority are quantifiably additive, not synergistic, in human patients (8). These observations suggest a need to reassess design principles for combination therapies.

Trials of ICI combinations in advanced urothelial cancer have had mixed results. This analysis leverages additivity models to reconcile these heterogeneous trial outcomes and illustrate several principles for understanding the success or failure of new combination therapies. First, the remarkable OS benefit of enfortumab vedotin plus pembrolizumab (HR 0.47) demonstrates that additivity does not imply modest efficacy; transformative regimens can be made from additive combinations of individually powerful agents (13). Second, the DANUBE trial shows that additivity does not ensure benefit: a drug with marginal single-agent activity predictably failed to improve outcomes (12). Third, the clinical efficacy of combination therapies can be largely predicted from single-agent efficacy, providing a framework to optimize future trials and drug development. An exception arose in trials with investigator’s choice of platinum, in which ICIs were additive with cisplatin but exhibited less-than-additive PFS with carboplatin. Because these analyses are in reference to each platinum’s own PFS, this does not merely reflect cisplatin’s intrinsically superior efficacy. Rather, this provides significant clinical evidence that carboplatin blunts the potential benefit of ICIs, supporting the preferential use of cisplatin alongside ICIs in this setting. Whether this platinum-specific interaction extends to other cancers warrants future study. This clinical evidence of platinum-specific drug interactions is consistent with recently reported translational studies reporting different immunologic effects of cisplatin and carboplatin (24) and helps explain why CheckMate 901, which exclusively used cisplatin, demonstrated a survival benefit, whereas IMvigor130 and KEYNOTE-361, which allowed carboplatin, did not (9–11).

The additive effects of contemporary ICI-based combinations for metastatic urothelial cancer raise the question of whether sequential therapy could achieve similar benefits. Hypothetically, if scans could detect impending failure and therapies were switched just before progression, then additive PFS would suggest upfront combination (A + B) or sequencing (A→B) could yield similar OS, assuming comparable tolerability. In practice, such foresight is rarely possible, and the aggressive nature of metastatic urothelial cancer often leads to clinical decline at progression; a large observational study found that only 43% of patients who received first-line therapy could receive a second-line therapy (25). This likely explains why additive combinations can improve OS over sequential use, even without synergy. The strongest argument for first-line combinations may therefore be pragmatic: many patients will not have a second chance at treatment. There are related implications for biomarkers. In theory, predictive biomarkers could reduce the need for combination therapy by guiding sequential strategies; for example, identifying patients likely to durably respond to ICI alone. However, it remains uncertain whether sufficiently accurate biomarkers can be developed to replace combination therapy in addressing interpatient heterogeneity.

Our study has potential limitations. First, we imputed IPD from published Kaplan–Meier curves and at-risk tables because original IPD are unavailable. However, this approach is widely used and combines the practical benefits of trial-level meta-analyses with the power of IPD meta-analyses (8, 26–30). Because the additivity model relies on Kaplan–Meier distributions, accurately reconstructed Kaplan–Meier distributions are sufficient to test for non-additive interaction. Second, for some drugs, monotherapy PFS was only available in the relapsed/refractory setting, as novel single-agent trials are seldom ethical in the first-line setting. For pembrolizumab, first- and second-line monotherapy data were available, and a sensitivity analysis found that choice of dataset did not change conclusions. Moreover, if a drug is more active at first-line therapy, then basing predictions on second-line data may underestimate the efficacy of first-line combination therapy, creating a bias to claim synergy where none exists. Therefore a finding of “no synergy” is robust to the use of second-line data. Third, the scope of these results is limited to the metastatic setting. While additivity models could in principle be adapted to adjuvant/neoadjuvant settings, clinical data on new single agents are often limited to the relapsed/refractory metastatic setting. If perioperative outcomes were to be predicted from drug activity in metastatic disease, it would be difficult to discern whether deviations from additivity were attributable to drug interactions or to differences in drug efficacy between adjuvant/neoadjuvant patients versus relapsed/refractory metastatic patients, which almost certainly exist. Fourth, although patient characteristics were broadly similar across studies (Supplementary Table S1), second-line monotherapy cohorts tended to have more advanced disease, which again can bias our analysis to detect synergy where none exists. For example, patients treated with pembrolizumab in KEYNOTE-045 had more visceral metastases (89%) than patients in EV-302 (72%) or KEYNOTE-361 (74%), and visceral metastases in urothelial cancer are associated with shorter responses to pembrolizumab (31). This difference could underestimate the first-line efficacy of pembrolizumab, causing an actually additive combination to be mistakenly identified as synergistic. The absence of detectable synergy despite this bias strengthens our conclusions. Ideally all comparisons would be drawn from three-arm trials (A, B, and A + B), but such designs are often infeasible for ethical reasons. Fifth, modeling of long-term outcomes is limited by follow-up of single-agent datasets. For example, the median follow-up of single-agent enfortumab vedotin was 15 months, limiting the additivity model to this timeframe. Sixth, although antitumor efficacy is demonstrably predictable by additivity models (8), different approaches may be better suited to predicting adverse events (32).

Our analyses address PFS not OS; OS is influenced by subsequent therapies, cross-over, and post-progression survival, details of which are often unreported or confounded (8). Combination therapies could improve or not improve OS for reasons unrelated to drug interaction. Although time to first progression does not capture benefits of subsequent therapies, it provides a clear test of interactions at the first line. A model of OS-based additivity could be valuable but would require assumptions about post-progression therapies that vary across trials. Some insights can be drawn by considering results of this study in context of OS endpoints of these trials. The studied combinations, except those containing carboplatin, produced additive PFS distributions. Such additive PFS could only yield OS synergy if the combination enhances sensitivity to post-progression therapies. Such “collateral sensitivity” could have intriguing implications for treatment algorithms. Among the combinations examined, only enfortumab vedotin plus pembrolizumab (EV-302) and nivolumab plus gemcitabine–cisplatin (CheckMate 901) significantly improved OS in the intention-to-treat population. EV-302 reported a PFS HR of 0.45 and OS HR of 0.47, whereas CheckMate 901 reported a PFS HR of 0.72 and OS HR of 0.78. Although comparing PFS and OS HRs across trials has limitations, the similar reductions in risk of disease progression and death for these two regimens does not suggest that they meaningfully enhance sensitivity to post-progression therapies.

We sought to learn from recent successes and failures in phase III trials in first-line treatment of metastatic urothelial cancer to determine how we should construct future combination regimens. We demonstrate that choice of cytotoxic therapy has a significant impact on the efficacy of ICI-containing combination therapies and that trial results can largely be explained by drug additivity. Our results are not a condemnation of research on drug synergy; however, synergy has been neither necessary, sufficient, nor common for improving clinical outcomes (8). Transformative advances such as enfortumab vedotin plus pembrolizumab can arise from additive combinations of individually effective agents. Given the rarity of synergy, limited resources, and vast number of possible combination regimens to explore in clinical trials, combining the most active, non–cross-resistant agents may be the most efficient and effective strategy to further improve cancer treatment.

## Supplementary Material

Supplementary Figure 1 Multiplying Hazard Ratios does not produce a specific prediction for combination therapy PFS

Supplementary Figure 2 Additional monotherapy PFS curves used for predictions

Supplementary Figure 3 Comparison of Progression-Free Survival (PFS) curves obtained from Kaplan-Meier fitting of imputed individual patient data (IPD) and digitized PFS curves from trial publications

Supplementary Figure 4 Graphical representation of workflow for predicting Progression-Free Survival of combination therapies

Supplementary Figure 5 Comparison of predicted enfortumab vedotin + pembrolizumab PFS with observed combination PFS from the EV-302 and EV-103 Cohort K studies

Supplementary Figure 6 Predictions of combination therapy PFS for patients who received cisplatin vs carboplatin

Supplementary Figure 7 Predictions of pembrolizumab combination efficacy using KEYNOTE-361 pembrolizumab monotherapy data

Supplementary Data 1 Digitized Kaplan-Meier curves and imputed individual patient data from all trial arms analyzed

Supplementary Table 1 Summary of trial details and patient charactertistics for investigated trials

## Data Availability

Kaplan–Meier distributions and imputed IPD of all data used in this study are provided in Supplementary Data S1.
