# Supplementary material for: Additivity, Not Synergy, Underlies the Efficacy of Current Combination Regimens in Urothelial Cancer
Source: Cancer Res Commun. 2026 Jun 19;6(6):1447–54. doi: 10.1158/2767-9764.CRC-26-0157 (PMC13280896; doi:10.1158/2767-9764.CRC-26-0157)
Supplement: Supplementary Figure 1 — Multiplying Hazard Ratios does not produce a specific prediction for combination therapy PFS [file crc-26-0157_supplementary_figure_1_suppsf1.pdf]

Supplementary Figure 1

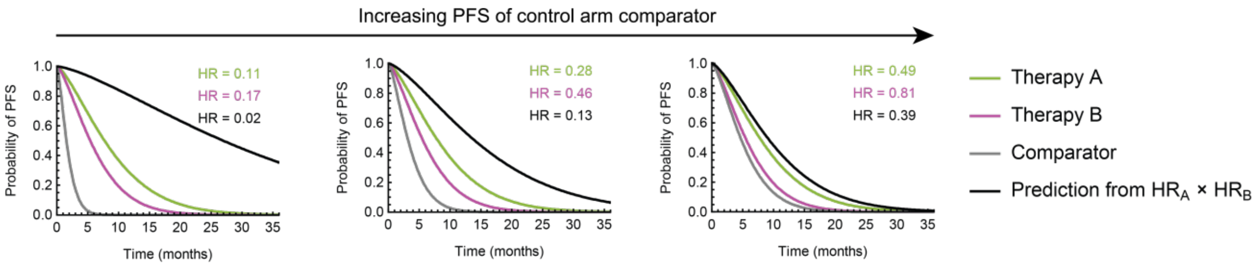

Supplementary Figure 1 | Combination therapy Progression-Free Survival (PFS) predicted by hazard ratio multiplication for a combination of Therapy A + Therapy B. Predictions shown with increasing PFS of control arm comparators from left to right. Increasing PFS of control arm comparator (thereby increasing hazard ratio between PFS of comparator and Therapies A and B) results in progressively worse predicted combination therapy PFS. In each plot, Hazard Ratios are obtained by comparing PFS curves for Therapy A, Therapy B, and predicted Therapy A + Therapy B to the control arm comparator via Cox Proportional Hazards.
