# Supplementary material for: Additivity, Not Synergy, Underlies the Efficacy of Current Combination Regimens in Urothelial Cancer
Source: Cancer Res Commun. 2026 Jun 19;6(6):1447–54. doi: 10.1158/2767-9764.CRC-26-0157 (PMC13280896; doi:10.1158/2767-9764.CRC-26-0157)
Supplement: Supplementary Figure 2 — Additional monotherapy PFS curves used for predictions [file crc-26-0157_supplementary_figure_2_suppsf2.pdf]

Supplementary Figure 2

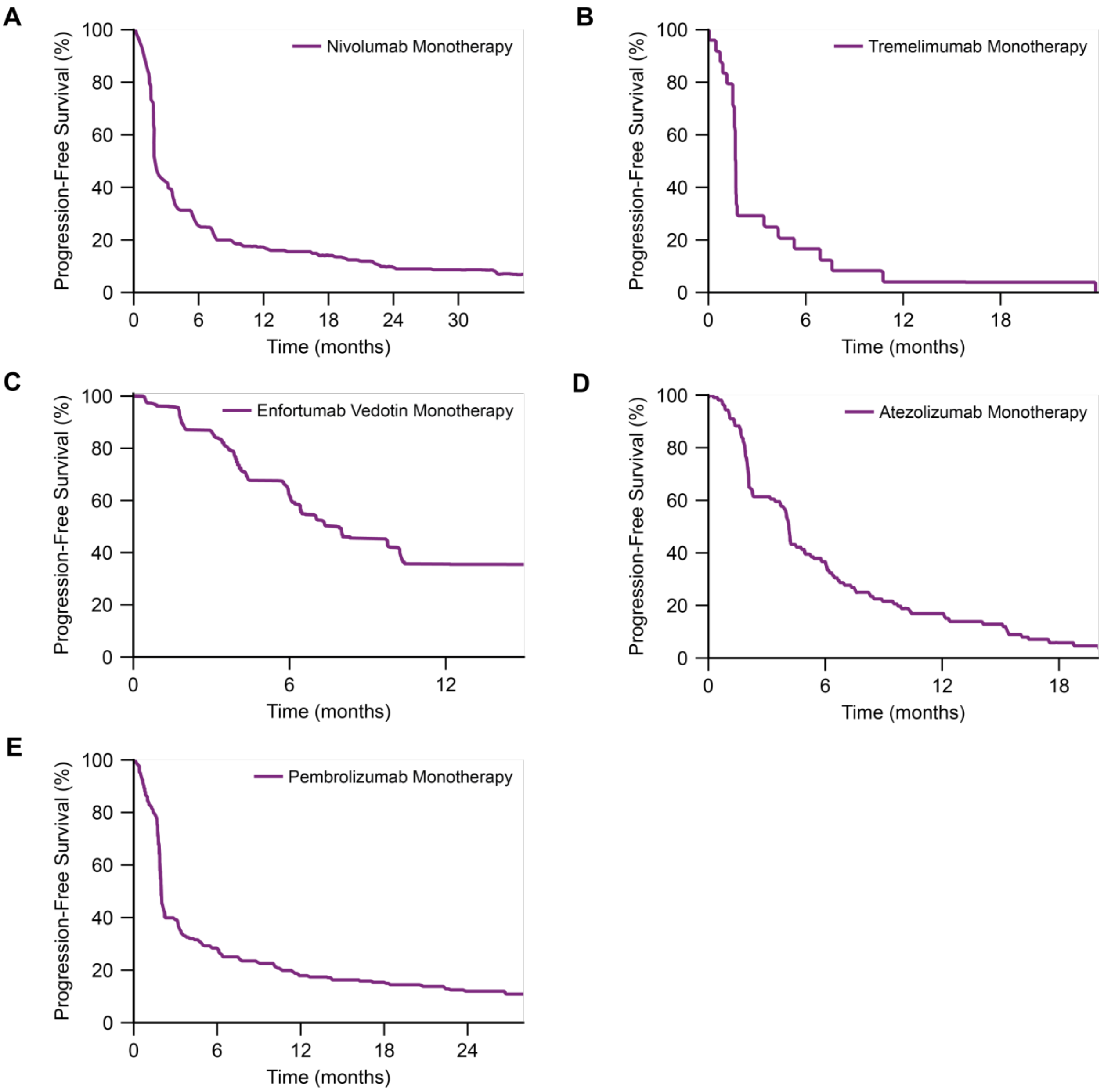

Supplementary Figure 2 | Additional PFS curves used for predictions. Monotherapy PFS curves not shown in main text used for prediction of combination therapy PFS. PFS curves obtained from the following trials: A. CheckMate 275. B. HCRN GU 17-294. C. EV-103. D. IMvigor 211. E. KEYNOTE-045.
