# Supplementary material for: Additivity, Not Synergy, Underlies the Efficacy of Current Combination Regimens in Urothelial Cancer
Source: Cancer Res Commun. 2026 Jun 19;6(6):1447–54. doi: 10.1158/2767-9764.CRC-26-0157 (PMC13280896; doi:10.1158/2767-9764.CRC-26-0157)
Supplement: Supplementary Figure 3 — Comparison of Progression-Free Survival (PFS) curves obtained from Kaplan-Meier fitting of imputed individual patient data (IPD) and digitized PFS curves from trial publications [file crc-26-0157_supplementary_figure_3_suppsf3.pdf]

Supplementary Figure 3

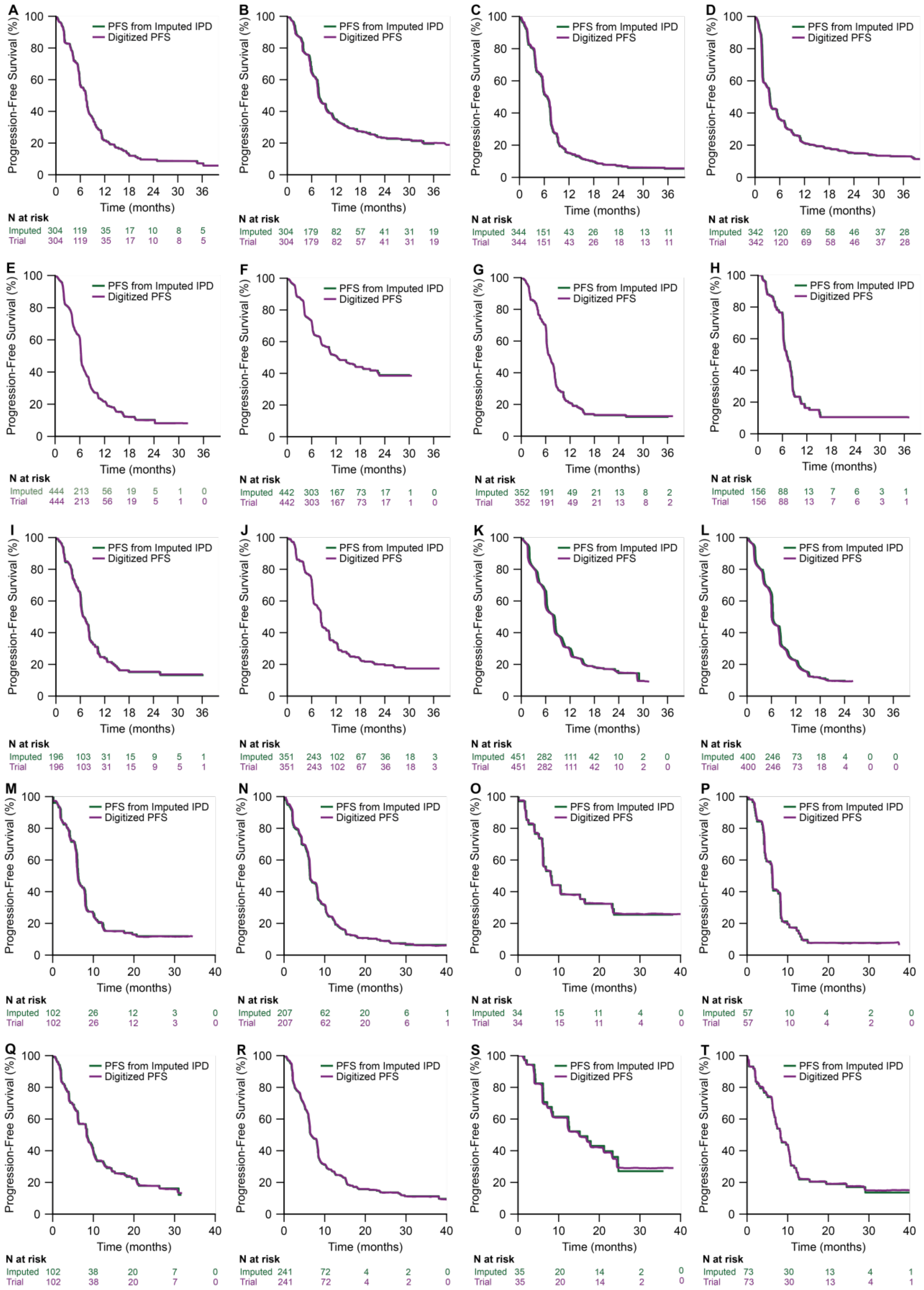

Supplementary Figure 3 | Comparison of Progression-Free Survival (PFS) curves obtained from Kaplan-Meier fitting of imputed individual patient data (IPD) and digitized PFS curves from trial publications. **A)** CheckMate 901, gemcitabine+cisplatin. **B)** CheckMate 901, nivolumab+gemcitabine+cisplatin **C)** DANUBE, gemcitabine+cisplatin/carboplatin. **D)** DANUBE, durvalumab+tremelimumab. **E)** EV-302, gemcitabine+cisplatin/carboplatin. **F)** EV-302, enfortumab-vedotin+pembrolizumab. **G)** KEYNOTE-361, gemcitabine+cisplatin/carboplatin. **H)** KEYNOTE-361, gemcitabine+cisplatin only subgroup. **I)** KEYNOTE-361, gemcitabine+carboplatin only subgroup. **J)** KEYNOTE-361, pembrolizumab+gemcitabine+cisplatin/carboplatin. **K)** IMvigor130, atezolizumab+gemcitabine+cisplatin/carboplatin. **L)** IMvigor130, gemcitabine+cisplatin/carboplatin. **M)** IMvigor130, gemcitabine+cisplatin only, IC 0/1 subgroup. **N)** IMvigor130, gemcitabine+carboplatin only, IC 0/1 subgroup. **O)** IMvigor130, gemcitabine+cisplatin only, IC 2/3 subgroup. **P)** gemcitabine+carboplatin only, IC 2/3 subgroup. **Q)** atezolizumab+gemcitabine+cisplatin only, IC 0/1 subgroup. **R**) atezolizumab+gemcitabine+carboplatin only, IC 0/1 subgroup. **S)** atezolizumab+gemcitabine+cisplatin only, IC 2/3 subgroup. **T)** atezolizumab+gemcitabine+carboplatin only, IC 2/3 subgroup.
