# Supplementary material for: Additivity, Not Synergy, Underlies the Efficacy of Current Combination Regimens in Urothelial Cancer
Source: Cancer Res Commun. 2026 Jun 19;6(6):1447–54. doi: 10.1158/2767-9764.CRC-26-0157 (PMC13280896; doi:10.1158/2767-9764.CRC-26-0157)
Supplement: Supplementary Figure 4 — Graphical representation of workflow for predicting Progression-Free Survival of combination therapies [file crc-26-0157_supplementary_figure_4_suppsf4.pdf]

Supplementary Figure 4

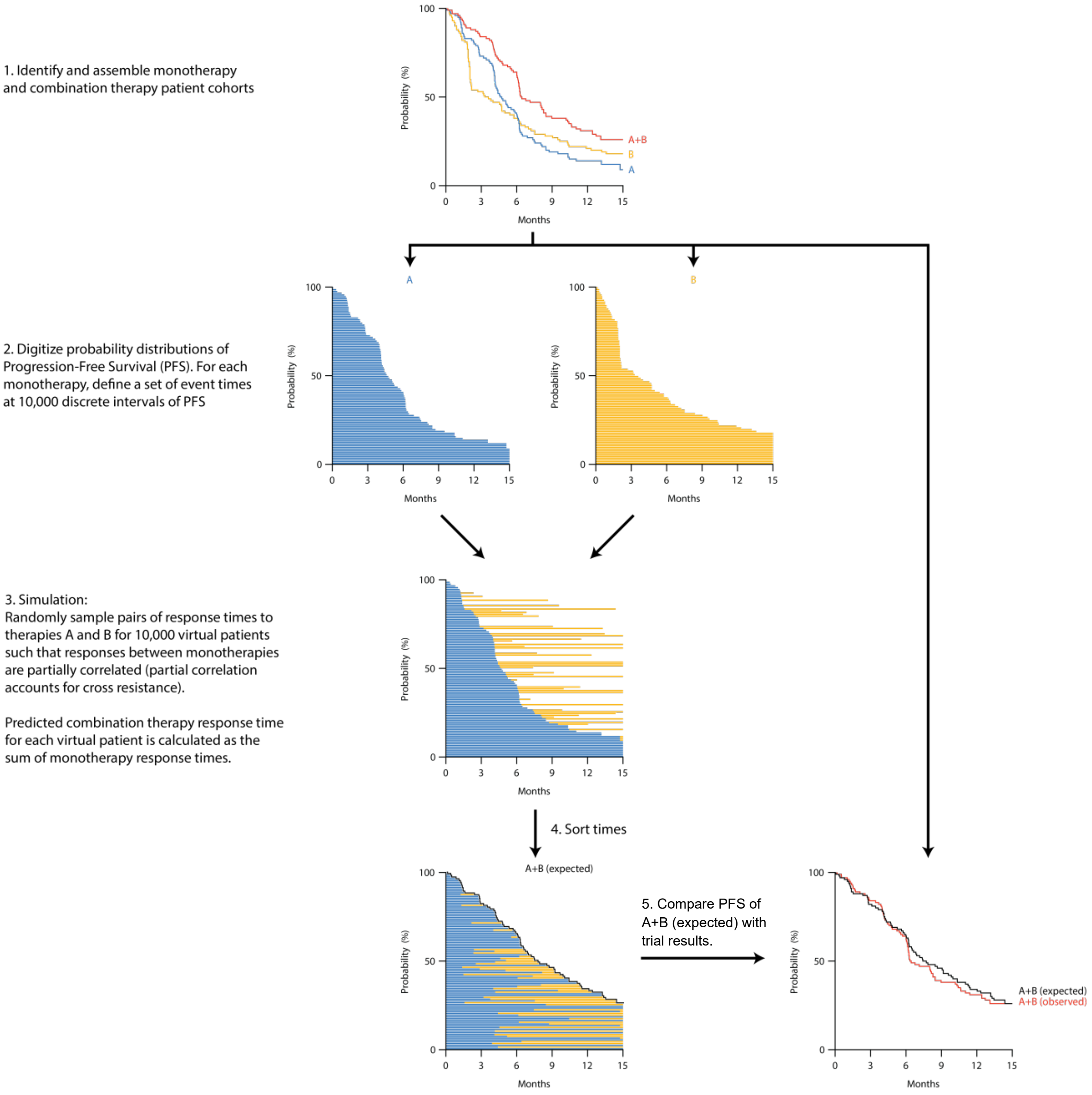

Supplementary Figure 4 | Graphical representation of workflow for prediction of Progression-Free Survival (PFS) for combination therapy A+B, given PFS distributions for A and B alone.
