# Supplementary material for: Additivity, Not Synergy, Underlies the Efficacy of Current Combination Regimens in Urothelial Cancer
Source: Cancer Res Commun. 2026 Jun 19;6(6):1447–54. doi: 10.1158/2767-9764.CRC-26-0157 (PMC13280896; doi:10.1158/2767-9764.CRC-26-0157)
Supplement: Supplementary Figure 5 — Comparison of predicted enfortumab vedotin + pembrolizumab PFS with observed combination PFS from the EV-302 and EV-103 Cohort K studies [file crc-26-0157_supplementary_figure_5_suppsf5.pdf]

Supplementary Figure 5

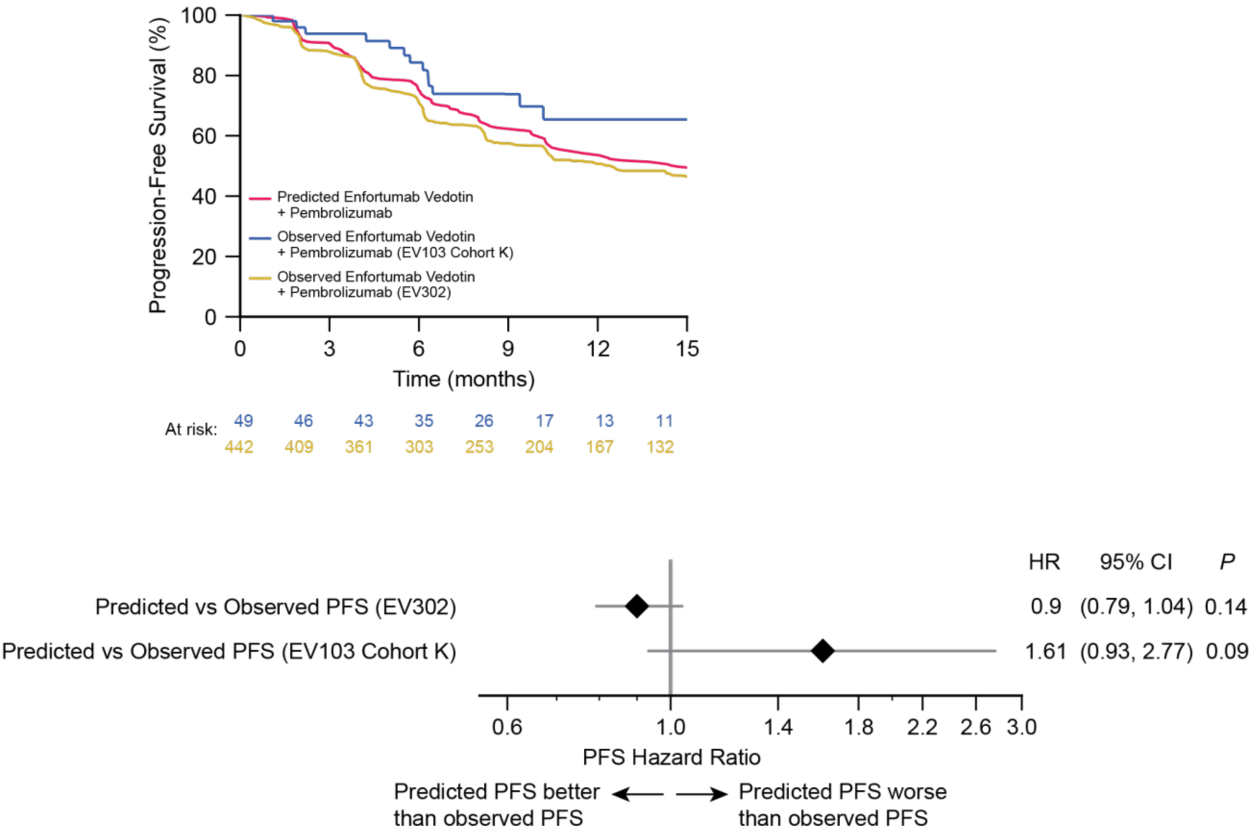

Supplementary Figure 5 | Comparison of predicted enfortumab vedotin + pembrolizumab PFS (red) with observed combination PFS from the phase 3 EV-302 (yellow) and phase 2 EV-103 Cohort K (blue) studies. Forest plot compares observed PFS in each study with predicted combination PFS under additivity; a hazard ratio of less than 1 indicates predicted PFS was superior to observed PFS and a hazard ratio of greater than 1 indicates predicted PFS was inferior to observed PFS. Predicted and observed PFS distributions were compared using Cox proportional hazards.
