# Supplementary material for: Additivity, Not Synergy, Underlies the Efficacy of Current Combination Regimens in Urothelial Cancer
Source: Cancer Res Commun. 2026 Jun 19;6(6):1447–54. doi: 10.1158/2767-9764.CRC-26-0157 (PMC13280896; doi:10.1158/2767-9764.CRC-26-0157)
Supplement: Supplementary Figure 6 — Predictions of combination therapy PFS for patients who received cisplatin vs carboplatin [file crc-26-0157_supplementary_figure_6_suppsf6.pdf]

Supplementary Figure 6

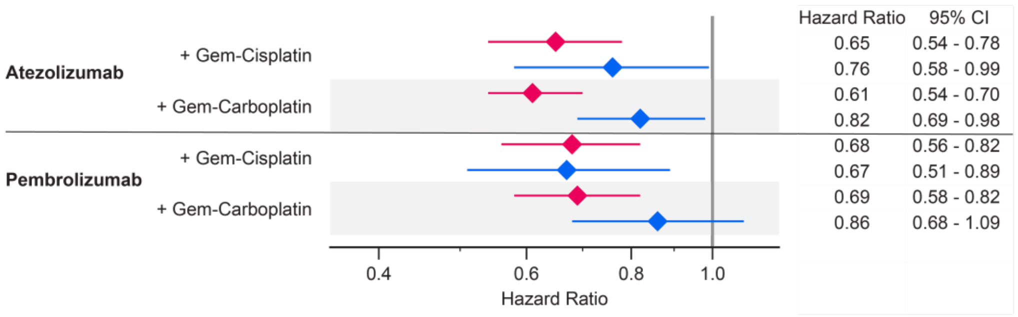

Supplementary Figure 6 | Predictions of combination therapy PFS for patients who received cisplatin vs carboplatin. Forest plot of predicted (red) and observed (blue) combination therapy hazard ratios in choice of cisplatin and choice of carboplatin patients for both atezolizumab (top) and pembrolizumab (bottom). Predictions of combination therapy PFS for cisplatin-containing combinations were made using PFS curves for patients who received gemcitabine-cisplatin in IMvigor 130 and KEYNOTE-361, and similarly for carboplatin. Predicted hazard ratios and confidence intervals obtained by comparing predicted combination therapy PFS to imputed control arm individual patient data using Cox proportional hazards.
