# Supplementary material for: Additivity, Not Synergy, Underlies the Efficacy of Current Combination Regimens in Urothelial Cancer
Source: Cancer Res Commun. 2026 Jun 19;6(6):1447–54. doi: 10.1158/2767-9764.CRC-26-0157 (PMC13280896; doi:10.1158/2767-9764.CRC-26-0157)
Supplement: Supplementary Figure 7 — Predictions of pembrolizumab combination efficacy using KEYNOTE-361 pembrolizumab monotherapy data [file crc-26-0157_supplementary_figure_7_suppsf7.pdf]

Supplementary Figure 7

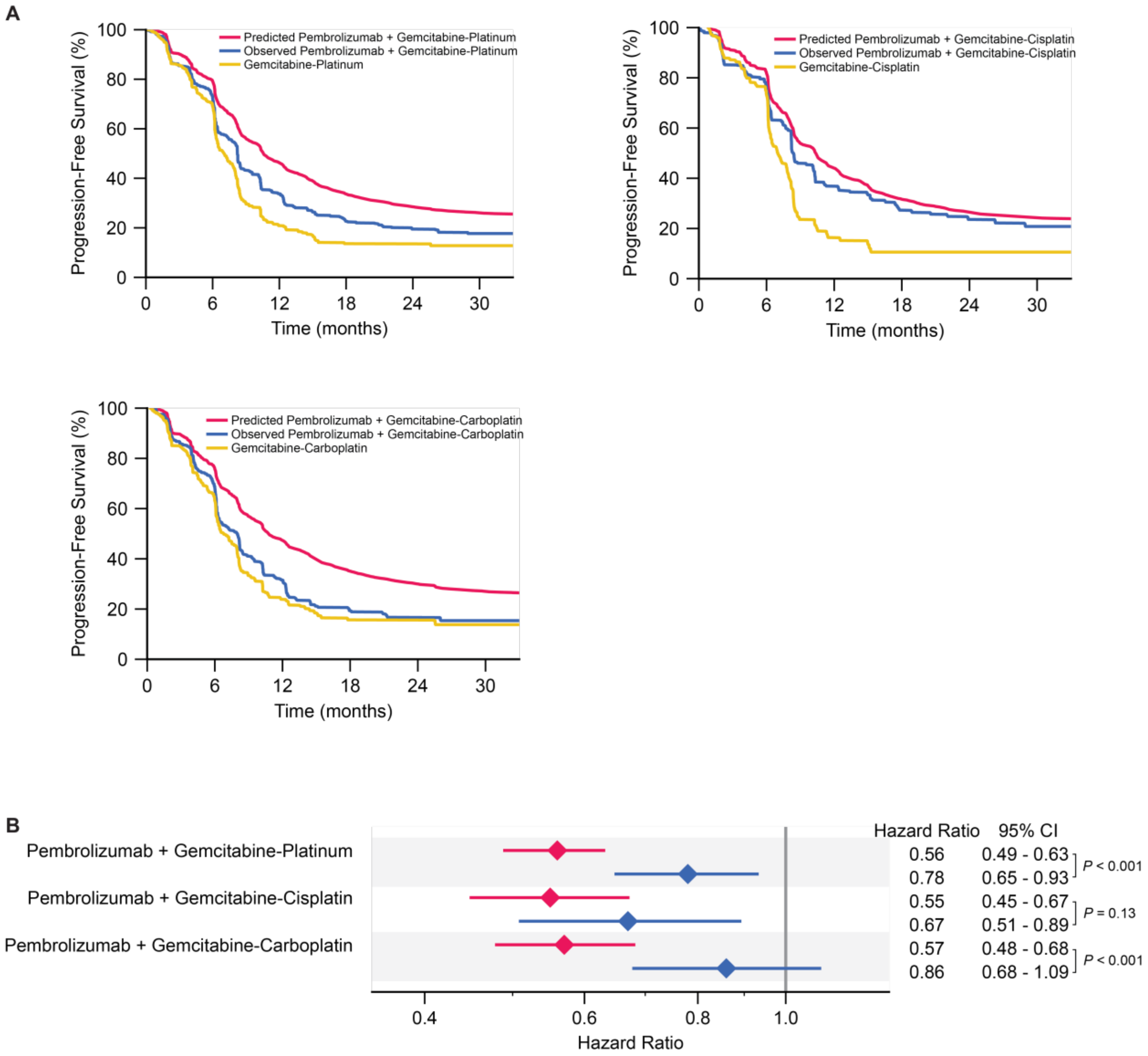

Supplementary Figure 7 | Predictions of pembrolizumab combination efficacy using KEYNOTE-361 pembrolizumab monotherapy data. **A.** Predictions of pembrolizumab + gemcitabine-platinum combination efficacy as well as platinum-specific predictions of pembrolizumab + gemcitabine-cisplatin and pembrolizumab + gemcitabine-carboplatin combination efficacy. Predictions made using KEYNOTE-361 pembrolizumab monotherapy. **B.** Forest plot comparing hazard ratios of predicted (red) and observed (blue) pembrolizumab + gemcitabine-platinum vs gemcitabine-platinum in ITT patients, choice of cisplatin patients, and choice of carboplatin patients. Predicted hazard ratios and confidence intervals obtained by comparing predicted combination therapy PFS to imputed control arm individual patient data using the Cox proportional hazards model. P values obtained by comparing expected and observed combination PFS (Cox proportional hazards model).
